# Laser-Visible Face Image Translation and Recognition Based on CycleGAN and Spectral Normalization

**DOI:** 10.3390/s23073765

**Published:** 2023-04-06

**Authors:** Mingyu Qin, Youchen Fan, Huichao Guo, Laixian Zhang

**Affiliations:** 1 Graduate School, Space Engineering University, Beijing 101416, China; 2 School of Space Information, Space Engineering University, Beijing 101416, China; 3 Department of Electronic and Optical Engineering, Space Engineering University, Beijing 101416, China

**Keywords:** image translation, range-gated, spectral normalization, face recognition, CycleGAN

## Abstract

The range-gated laser imaging instrument can capture face images in a dark environment, which provides a new idea for long-distance face recognition at night. However, the laser image has low contrast, low SNR and no color information, which affects observation and recognition. Therefore, it becomes important to convert laser images into visible images and then identify them. For image translation, we propose a laser-visible face image translation model combined with spectral normalization (SN-CycleGAN). We add spectral normalization layers to the discriminator to solve the problem of low image translation quality caused by the difficulty of training the generative adversarial network. The content reconstruction loss function based on the Y channel is added to reduce the error mapping. The face generated by the improved model on the self-built laser-visible face image dataset has better visual quality, which reduces the error mapping and basically retains the structural features of the target compared with other models. The FID value of evaluation index is 36.845, which is 16.902, 13.781, 10.056, 57.722, 62.598 and 0.761 lower than the CycleGAN, Pix2Pix, UNIT, UGATIT, StarGAN and DCLGAN models, respectively. For the face recognition of translated images, we propose a laser-visible face recognition model based on feature retention. The shallow feature maps with identity information are directly connected to the decoder to solve the problem of identity information loss in network transmission. The domain loss function based on triplet loss is added to constrain the style between domains. We use pre-trained FaceNet to recognize generated visible face images and obtain the recognition accuracy of Rank-1. The recognition accuracy of the images generated by the improved model reaches 76.9%, which is greatly improved compared with the above models and 19.2% higher than that of laser face recognition.

## 1. Introduction

With the improvement of computing power and the rapid development of computer vision, visible face recognition accuracy can reach more than 99% [1]. However, the recognition accuracy is greatly reduced or recognition is even impossible due to the poor quality of visible imaging under night conditions [2,3]. Some researchers propose to use near-infrared imaging systems, short-wave infrared imaging systems and other solutions to solve this problem [4,5,6]. Although these systems can be used at night, they cannot meet the requirements for long-distance and high-definition imaging due to the limited imaging distance. Range-gated laser imaging instrument uses lasers with high brightness, strong monochromaticity, and good directionality as active illumination sources [7]. It uses range gating technology to image the target at a specific distance, which can physically isolate the scattered signal beyond the target distance and can effectively suppress the backscattering interference of the laser [8]. Therefore, range-gated laser imaging can adapt to long-distance conditions at night, and the resulting images have the advantage of high resolution compared with other imaging systems. However, the laser image has a large modal difference from the visible image, which is not conducive to human observation. At the same time, most of the existing databases are visible images, and the effect of directly matching laser images with visible images is not good. Therefore, it is necessary to translate laser face images into visible face images, reconstruct visible faces according to laser faces, restore visible facial features, and improve the face visual effect and face recognition accuracy [9]. 

Image translation is the process of transforming source domain images into target domain images, that is, keeping the content characteristics of source domain images unchanged and converting the image according to the style characteristics of target domain images. In recent years, with the increasing application of generative adversarial networks in image translation, more and more researchers have explored the field of cross-model image translation and achieved good results. Zhang et al. [10] added a guide network at the end of the visible feature extraction to ensure the reconfigurability of the coded features and ensure that the reconstruction information contains semantic information. Wang et al. [11] proposed a network framework combining GAN and face feature point detector network to convert thermal images into visible images. Since there are almost no facial features in the thermal image, the generator is constrained by the facial feature point detection network to reduce the dislocation of facial features and improve the quality of image translation. Chen et al. [12] proposed converting thermal infrared images into visible images and recognizing translated visible faces. The author used the face parsing network to extract the semantic information of the face and constrain the generated face to improve face recognition accuracy. K.K. Babu et al. [13] proposed the PCSGAN framework for converting thermal infrared images into visible images, which improved the quality of the generated images by adding cyclic perception loss and synthetic perception loss. Mei et al. [6] used pre-trained StyleGAN2 [14] to learn visible images and employed the knowledge learned prior to convert Thermal images into visible images based on GAN. The current cross-domain image translation models are mainly intended for infrared, short-wave infrared, and thermal infrared image translation, but less so for laser image translation. These cross-domain image translation models have a reference role for laser-visible face image translation. Most methods enhance the generated image quality by adding a pre-trained network or adding loss functions to the framework of the GAN to constrain the generator. However, the number of laser datasets is small, and it is not easy to learn the correct mapping during training. Limited by the number of laser-visible face datasets, the above models are not suitable for laser-visible face translation. We need to improve the inherent shortcomings of generative adversarial networks to reduce the difficulty of training and improve the quality of generated images.

In this paper, the SN-CycleGAN model is designed for laser-visible face image translation, and a laser-visible face recognition framework based on feature retention is constructed. Subjective observation and objective quantification are used to evaluate the face translation results and recognition accuracy. In summary, our contributions are:

1. Laser-visible face image datasets. We analyze the laser-visible face image translation problem and acquire laser and visible images according to the experimental requirements. We analyze the characteristics of the acquired laser and visible images, then preprocess the laser and visible images, and finally establish the laser-visible face image datasets.
2. In the stage of laser face image translation, we design a discriminator combined with spectral normalization layers to enhance the stability of the network training, reduce mismapping of the generated images, and improve the quality of face image translation. The content reconstruction loss function based on Y channel is added to reduce the error mapping.
3. In the stage of laser face recognition, we propose a generator that can preserve the identity of face features. The shallow feature map in the encoder is added to the deep feature map in the decoder pixel by pixel to retain more face details and improve face recognition accuracy. A domain loss function based on triplet loss is added to constrain the style between the same domain.

## 2. Laser-Visible Face Image Dataset

At present, there are few publicly laser-visible face datasets available through the survey, so this paper uses self-built laser-visible face datasets. We first study the laser face to visible face image translation problem, and then clarify the face image acquisition requirements. We select image capture device and design image capture scenarios according to acquisition requirements. Finally, the image is preprocessed.

### 2.1. Self-Built Dataset

For the laser to visible face image translation problem, the dataset must meet two conditions [15].

1. Multi-modality: The dataset should contain laser face and visible face images. When solving the image translation task, the model searches for hidden correspondence between laser face and visible face images by training images from the two domains. If there is only a single image domain, the network cannot be trained to complete cross-domain image translation. In the test, it is difficult to determine the image translation result.
2. Matching: Laser images and visible images have the same attitude and angle so that the dataset can be used not only for supervised networks but also for unsupervised networks. It is required that laser and visible image capture devices have a certain degree of synchronization to obtain the image of the same target at the same time.

**Figure 1:**
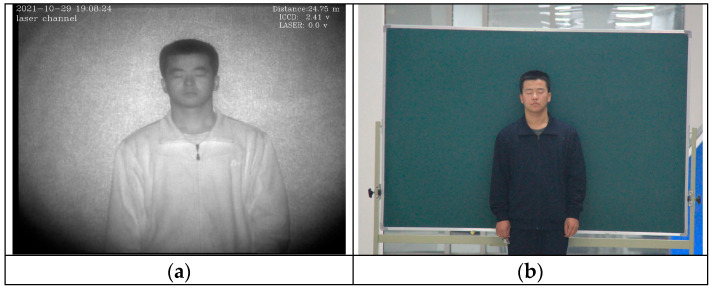
Laser and visible images. (**a**) Laser image. (**b**) Visible image.

We select a long corridor with controlled lighting as the collection location. We acquired laser images when the light is turned off and visible light images when the light is on. The visible acquisition device is placed close to the laser acquisition device and placed on it to reduce the difference caused by the different lens positions. The target is located at 26.5 m of the device. The acquisition equipment for visible images is a Canon 60D SLR camera, and the acquisition equipment for laser images is a range-gated laser imaging instrument developed by the laboratory [9]. The range-gated laser imaging instrument uses an 860 nm laser as an illumination source, which can emit a signal with high pulse energy, and at the same time can overcome the backscattering of laser active imaging to obtain high-resolution images under long-distance dark conditions. The laser module used in the range-gated laser imaging instrument has high energy, and the human eye hazard distance is 78.15 m without considering attenuation and system interference. Therefore, the target is required to close their eyes during acquisition to avoid damage to the target’s eyes [16]. We collected a total of 100 targets and obtained frontal face images. The acquired laser image and visible image are shown in Figure 1, and image parameters are shown in Table 1.

### 2.2. Data Preprocessing

The two devices have different fields of view causing laser images to be widened compared to visible images. In laser and visible images, the background occupies most of the frame, and the face occupies a smaller proportion than the whole picture. These backgrounds have no effect on the face image translation task. Therefore, the first step is to resize the laser image, the second step is to cut the laser and visible heads, and the third step is to remove the background of the laser and visible images.

The resolution of laser images is changed from 1024 × 768 to 893 × 768, and deformed laser images are more in line with the normal face size. Due to the poor accuracy of laser face detection by the existing face detection algorithm, the face is marked by the image labeling tool LabelImage, and then the marked face is cut to obtain the avatar with the background. At the same time, when labeling, we set the aspect ratio of the label box to 1:1 to prevent face deformation when resizing images. the background of the cut image is removed through the already trained segmentation model. Since the input and output of the segmentation model are three-channel images, the laser image becomes a three-channel image after segmenting the model, a three-channel image superimposed by three single-channel laser images.

**Figure 2:**
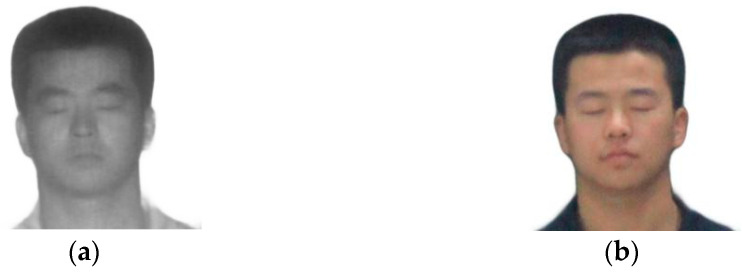
Data preprocessing results. (**a**) Laser face image. (**b**) Visible face image.

The laser image and the visible image are resized to 3 × 256 × 256, as shown in Figure 2. The 100 targets are divided into training and test sets in a 9:1 ratio, and datasets are doubled by mirror flipping. The final effective laser and visible training sets are 182 and 184 shots, respectively, and the laser and visible test sets are 26 and 26 shots, respectively.

## 3. Laser-Visible Face Image Translation

### 3.1. CycleGAN

The laser-visible face image datasets have two characteristics:

1. Laser face image and visible face image have no matching alignment, it is a non-matching dataset.
2. The preprocessed laser and visible images only have the avatar, and the scene is relatively single.

We chose CycleGAN [17] as the backbone network based on the above two points. As an unsupervised algorithm, CycleGAN is suitable for unmatched datasets, especially datasets where the image contours of the two domains do not change greatly.

**Figure 3:**
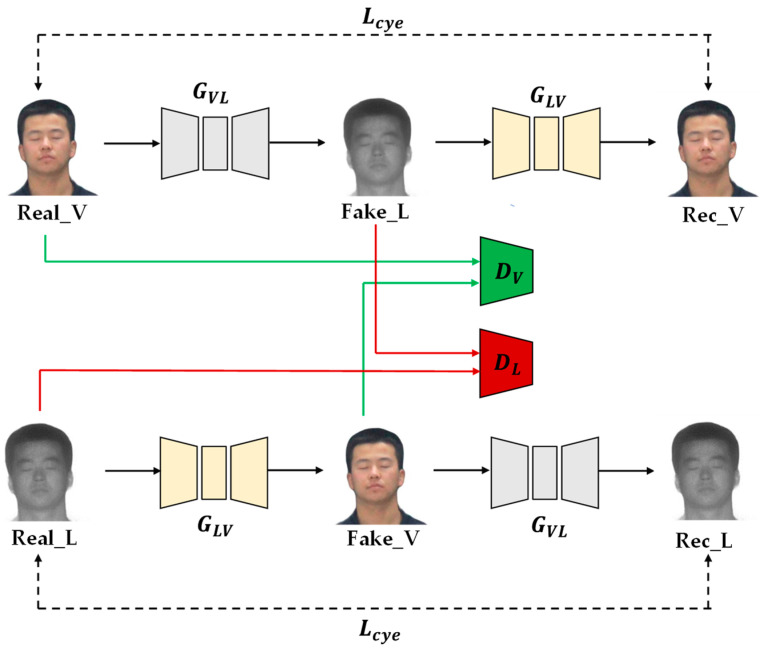
Structure of CycleGAN model.

Cyc1eGAN is an unsupervised image translation framework proposed by Zhu et al. It consists of two mirror links, each of which includes two generators and a discriminator. Figure 3 shows the model structure of CycleGAN. The generator $G_{VL}$ translates a visible image into a laser image, and the generator $G_{LV}$ translates a laser image into a visible light image. Discriminators are used to determining whether the input image is real or generated. At the same time, a cycle loss function is introduced to ensure that the content of the input image and the reconstructed image are consistent.

**Figure 4:**
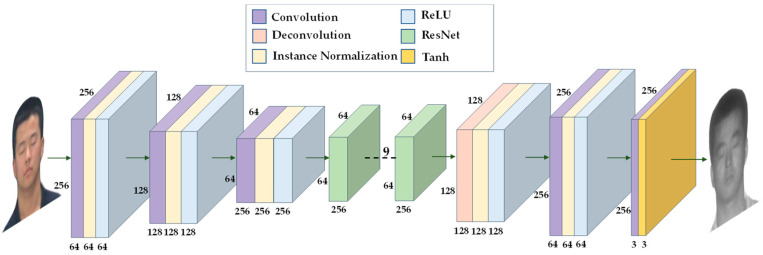
The architecture of the generator in the CycleGAN.

**Figure 5:**
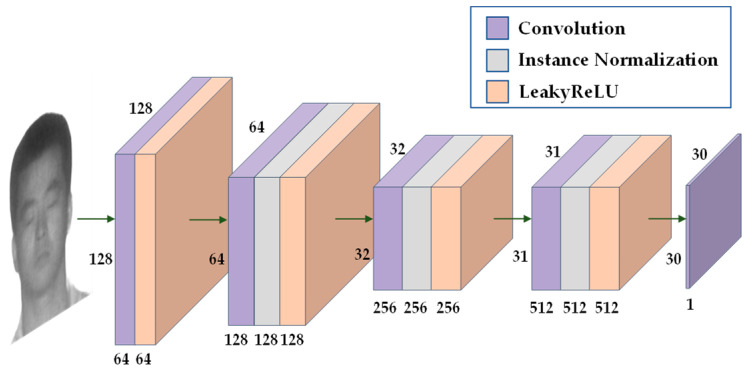
The architecture of the discriminator in the CycleGAN.

The generator consists of three parts: encoder, feature converter and decoder. The generator structure is shown in Figure 4. The encoder and decoder perform downsampling and upsampling operations, respectively, and the feature extractor uses nine residual modules. The residual module solves the problem of network degradation, ensures efficient gradient delivery, and improves the performance of the network to a certain extent. The discriminator uses PatchGAN [18], and the discriminator structure is shown in Figure 5. PatchGAN outputs a feature map of 30 × 30, which is different from the discriminator of GAN that outputs an evaluation value. Each pixel in the feature map represents a 70 × 70 area in the input image, allowing the discriminator to focus on more information.

### 3.2. SN-CycleGAN

CycleGAN has great shortcomings in laser-visible face image translation. First, CycleGAN uses GAN [19] as the basic network and has the same problems as GAN during network training, that is training difficulties, gradient disappearance, and model collapse [20]. When the discriminator does not converge, the discriminator cannot provide effective and correct feedback to the generator. The generator considers this to be a signal of it producing a good image, but the actual image quality is low. Second, the cycle consistency loss function uses the entire image as input, constraining not only the color information of the image but also the structural information of the image. The network is prone to learning error messages. We modify the network based on these two points. First, we improve the discriminator to improve the stability of network training. Second, we introduce the content reconstruction loss function based on the Y channel, which enhances the generator’s attention to image content and structure and improves the quality of the generated image.

#### 3.2.1. Discriminator Combined with Spectral Normalization

To solve the problem of GAN training difficulties, WGAN [20] uses Wasserstein distance instead of JS divergence in GAN, which transforms the solution problem of Wasserstein distance into an optimal solution problem for solving Lipschitz continuity. It requires the discriminator to satisfy the 1-Lipschitz constraint to eliminate the convergence problem in GAN training and make the training more stable. However, WGAN uses gradient clipping to directly limit the elements in the parameter matrix and does not allow them to exceed the fixed constant C. This method destroys the proportional relationship between the parameters.

The spectral normalization constraint proposed by SNGAN [21] is a method that satisfies the continuity of 1-Lipschitz without destroying the matrix structure. The spectral normalization constraint is the performance of spectral norm on the discriminator, which makes the discriminator satisfy the 1-Lipschitz condition. The activation function in the discriminator already satisfies this condition. Therefore, if the convolutional layer in the discriminator satisfies this condition, the discriminator satisfies 1-Lipschitz continuity. Since convolution is equivalent to matrix multiplication, when the parameter *W* of each layer of the convolution kernel can satisfy 1-Lipschitz continuity, the convolutional layer can satisfy 1-Lipschitz continuity, so that the discriminator satisfies 1-Lipschitz continuity [22]. 

The specific goal of the operation is to divide the spectral norm of *W* by each update, and the spectral norm is the maximum singular value of the matrix *W*. The calculation formula of the parameter matrix after spectral normalization is as follows.

(1) $$W_{SN}=\frac{W}{\sigma (W)},\sigma (W)=\underset{h:h\neq 0}{\max}\frac{{\left\|Wh\right\|}_{2}}{{\left\|h\right\|}_{2}},$$

where *W* is the parameter matrix, *h* is the input, σ(W) is the spectral norm of the matrix *W*, $W_{SN}$ is the updated parameter matrix. First, the spectral norm of each layer matrix is calculated, and the matrix divides the spectral norm is the processed weight matrix.

**Figure 6:**
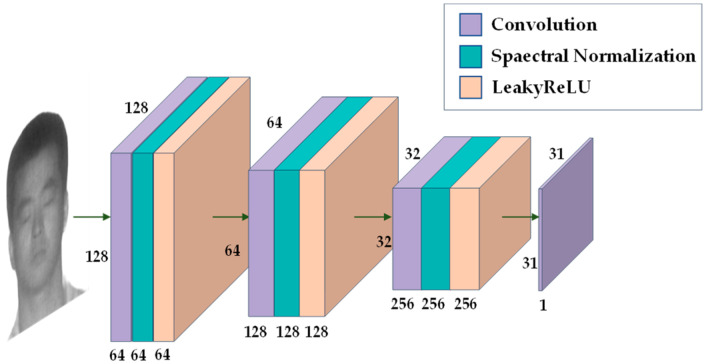
The architecture of the discriminator in the SN-CycleGAN.

The modified discriminator is shown in Figure 6. The first three convolutional layers of the discriminator are followed by spectral normalization layers and activation functions, and finally there is only one convolutional layer, which outputs a feature map of 31 × 31. Each pixel in the output feature map can represent a region in the input image, that is, the value of each pixel can determine the authenticity of the corresponding region. 

#### 3.2.2. Content Reconstruction Loss Function based on Y Channel

**Figure 7:**
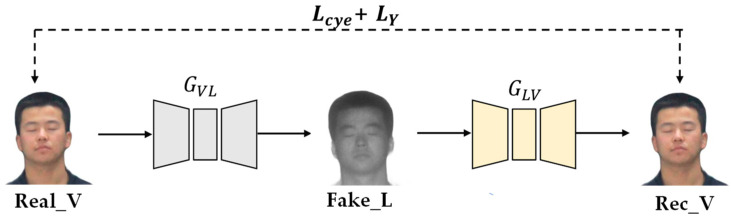
*L_Y_* loss function.

The content reconstruction loss function based on Y channel uses the L1 norm to calculate the distance between the real image and the reconstructed image of the Y-channel image. This distance is minimized as a way to improve the quality of the generated image [23,24], as shown in Figure 7 as $L_{Y}$. The Y channel represents the intensity and brightness information of the image and retains a lot of image detail information. Compared with the color information, the human eyes are more sensitive to the brightness information of the image. In CycleGAN, the cycle consistency loss function not only constrains the color information of the image but also constrains the content and structure information so that the generator can easily learn the wrong mapping. By adding the content reconstruction loss function based on the Y channel, the network pays attention to the global information of the image, as well as effectively improves the learning ability of the image content and structure.

The content reconstruction loss function based on the Y channel is shown in Formula (2).

(2) $$L_{Y}(G_{LV},G_{VL})=E_{v∼Pdata(v)}{\left\|Y(v)-Y\{G_{LV}[G_{VL}(v)]\}\right\|}_{1},$$

where Y(·) represents the extraction of image Y channel information.

#### 3.2.3. Total Loss Function

The loss functions of SN-CycleGAN include adversarial loss, cycle consistency loss, identity loss, and content reconstruction loss function based on Y channel.

Adversarial loss function:

(3) $$L_{GAN}(G_{VL},D_{L},L,V)=E_{l∼Pdata(l)}[\log(D_{L}(l))]+E_{v∼Pdata(v)}[1-\log(D_{L}(G_{VL}(v)))],$$

(4) $$L_{GAN}(G_{LV},D_{V},L,V)=E_{v∼Pdata(v)}[\log(D_{V}(v))]+E_{l∼Pdata(l)}[1-\log(D_{V}(G_{LV}(l)))],$$

where *v* and *l* are the visible image and laser image, $G_{VL}$ and $G_{VL}$ are the generated laser image and visible image, and $E_{l∼Pdata(l)}$ and $E_{v∼Pdata(v)}$ are the expected of the laser image and the visible image, respectively.

(5) $$L_{cyc}(G_{VL},G_{LV})=E_{l∼Pdata(l)}[{\left\|G_{VL}(G_{LV}(l))-l\right\|}_{1}]+E_{v∼Pdata(v)}[{\left\|G_{LV}(G_{VL}(v))-v\right\|}_{1}],$$

where $G_{VL}(G_{LV}(l))$ and $G_{LV}(G_{VL}(v))$ are the reconstructed laser and visible image, respectively.

To improve network performance, an identity loss function [17] is added:

(6) $$L_{identity}(G_{VL},G_{LV})=E_{l∼Pdata(l)}[{\left\|G_{VL}(l)-l\right\|}_{1}]+E_{v∼Pdata(v)}[{\left\|G_{LV}(v)-v\right\|}_{1}],$$

where $G_{VL}(l)$ means to input image *l* into generator $G_{VL}$, $G_{LV}(v)$ means to input image *v* into the generator $G_{LV}$.

Total loss function:

(7) $$\begin{array}{cc} L(G_{VL},G_{LV},D_{V},D_{L}) & =L_{GAN}(G_{VL},D_{L},V,L)+L_{GAN}(G_{LV},D_{V},V,L)\\ \; & \;+\alpha L_{cyc}(G_{VL},G_{LV})+\beta L_{identity}(G_{VL},G_{LV})+\delta L_{Y}(G_{VL},G_{LV}) \end{array},$$

where *α*, *β* and *δ* are the weights of $L_{cyc},L_{identity}$ and $L_{Y}$, respectively. *α* and *β* use the weights in the original paper, and *δ* is confirmed in the experiment in Section 3.3.2.

### 3.3. Laser-Visible Face Translation Experiment 

We use laser-visible face datasets for training and testing while comparing the improved network with Pix2Pix [18], U-GAT-IT [25], StarGAN [26], UNIT [27] and GP-UNIT [28]. The evaluation methods are subjective evaluation methods and objective evaluation methods. Subjective evaluation mainly relies on the observation of the human eye to compare the difference between real visible images and generated visible images. The objective evaluation uses FID. FID extracts the feature vector of original images and generated images using the inception network, and represents the difference between two image domains by calculating the distance of between two feature vectors. The lower the FID value, the better the quality of the generated image. MSE, SSIM and PSNR are mainly used to evaluate pixel-by-pixel aligned images, and they are not suitable for unmatched laser-visible face datasets.

#### 3.3.1. Experimental Environment and Parameter Settings

The experimental hardware platform and software platform are shown in Table 2 below.

In model training, we use Adam optimizer, where $\beta_{1}=0.5$, $\beta_{2}=0.999$. The initial learning rate is 0.0002, the first 100 epochs are 0.0002, and the last 100 decay by 1% until it is 0.

#### 3.3.2. Comparison of Network Training Processes

**Figure 8:**
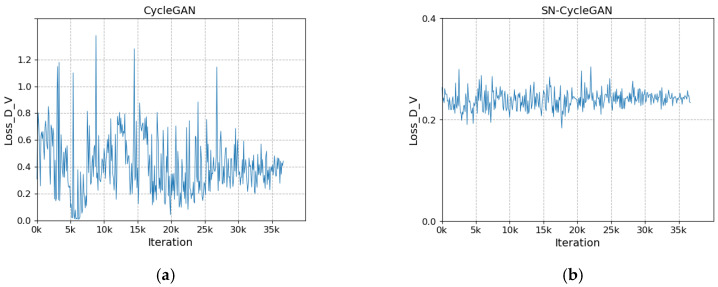
Comparison of discriminator loss for visible light. (**a**) CycleGAN, (**b**) SN-CycleGAN.

**Figure 9:**
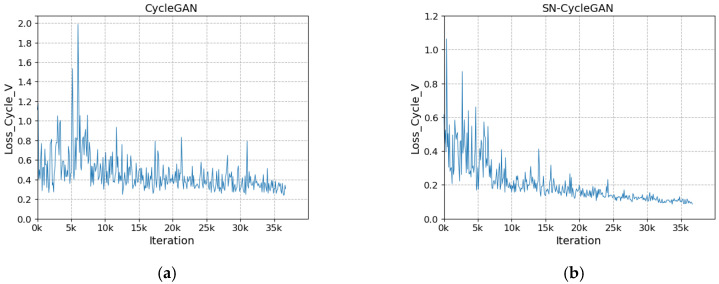
Comparison of cycle loss for visible light. (**a**) CycleGAN, (**b**) SN-CycleGAN.

**Figure 10:**
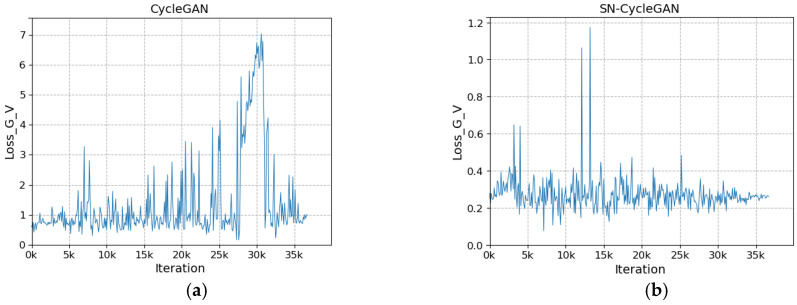
Comparison of generator loss for visible light. (**a**) CycleGAN, (**b**) SN-CycleGAN.

The network after modifying the discriminator is more stable during training. As shown in Figure 8a, with the increase in the number of iterations, the loss of the discriminator does not converge and fluctuates greatly. In Figure 8b, with the increase in the number of iterations, the discriminator loss gradually stabilizes and converges to about 0.23 in the fluctuation. In Figure 9a, the cycle loss of the visible image decreases slightly as the number of iterations increases, but it is not significant. In Figure 9b, with the increase in the number of iterations, the cycle loss of visible light shows a steady downward trend and gradually converges to about 0.1. In Figure 10a, as the number of iterations increases, the generator loss shows large and successive fluctuation. In Figure 10b, with the increase in the number of iterations, the cycle loss shows a steady downward trend and gradually converges to about 0.26. The stability of SN-CycleGAN during training is significantly improved compared with GAN, which has a role in improving the quality of the generated image.

#### 3.3.3. Weight Selection for Content Reconstruction Loss Function

In this experiment, the weight of the content reconstruction loss function in SN-CycleGAN is explored. We first test the weights over a larger range and then select the weights near the optimal weights for testing. We set the weights to 0, 5, 10, 15, 20 and 25, respectively, and as can be seen from Table 3, the value of FID is the smallest when the weight is 10. We selected 9 and 11 around 10 as the weights, and the FID values of the translated results were both maintained at 42. The trend for FID values is ‘M’ and FID at 10 is the minimum. Therefore, we choose 10 as the weight of the content reconstruction loss function.

#### 3.3.4. Comparison with Other Models

**Figure 11:**
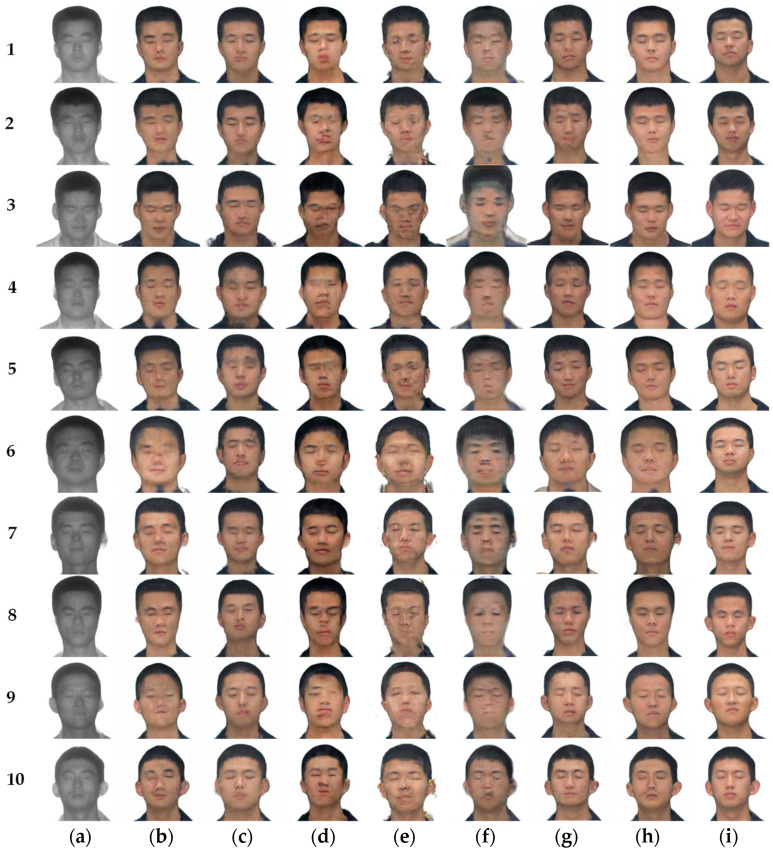
Different methods for Laser-visible facial image translation. (**a**) Laser image. (**b**) CycleGAN. (**c**) Pix2Pix. (**d**) UNIT. (**e**) UGATIT. (**f**) StarGAN. (**g**) DCLGAN. (**h**) Ours (SN-CycleGAN). (**i**) Ground truth.

SN-cycleGAN was compared with models such as CycleGAN, Pix2Pix, UNIT, UGATIT, StarGAN, and DLCGAN. In the experiment, all models used laser-visible face datasets. The parameters in the comparison frame are the parameters in the paper. The image translation results under different frameworks are shown in Figure 11, and the FID values are shown in Table 4.

In Figure 11, there are ten samples, represented by the numbers 1–10. The image of each row represents the same person. It can be seen from the figure that the translation results shown in Figure 11b maintain the face contour. However, some avatars have unclear boundaries between hair and face, and missing and distorted facial features. For example, the right eye disappears in picture Figure 11(b-6), and the left eye disappears in picture Figure 11(b-10). The images of Figure 11c are blurred on the whole, and the edge of the head appears jagged, partly because the image is not strictly aligned. The results of Figure 11d show that the five senses disappear and the five senses appear repeatedly. For example, the left eye of Figure 11(d-4) disappears, the right eye of Figure 11(d-9) disappears and the forehead and chin have red marks. The images in Figure 11e show overall ghosting, with clear features impossible to distinguish. The results in Figure 11f appear shaded on the face; the color of facial features is light, and the boundary line of face is not clear. The translation results in Figure 11g have problems with missing hairline boundaries and uneven skin tone, as well as black shadows on some of the face. Figure 11h facial details are more similar to real visible light images, and there is no distortion and blur of facial features.

The quantitative results show that the FID scores of the translated result of the proposed model are the lowest, decreasing by 16.902, 13.781, 10.056, 57.722, 62.598 and 0.761 compared with CycleGAN, Pix2Pix, UNIT, UGATIT StarGAN and DCLGAN, respectively. Although SN-CycleGAN is only 0.761 lower than DCLGAN, it is a subjectively better SN-CycleGAN.

From the subjective analysis, the translation results of the improved model do not show facial distortion or blurred facial features. There are almost no artifacts that affect facial features, and the facial features basically restored the reference image. The objective quantitative results show that the FID value of the translation results of the improved model is lower than that of other frameworks, and the translation results are of better quality. 

#### 3.3.5. Ablation Experiment

**Figure 12:**
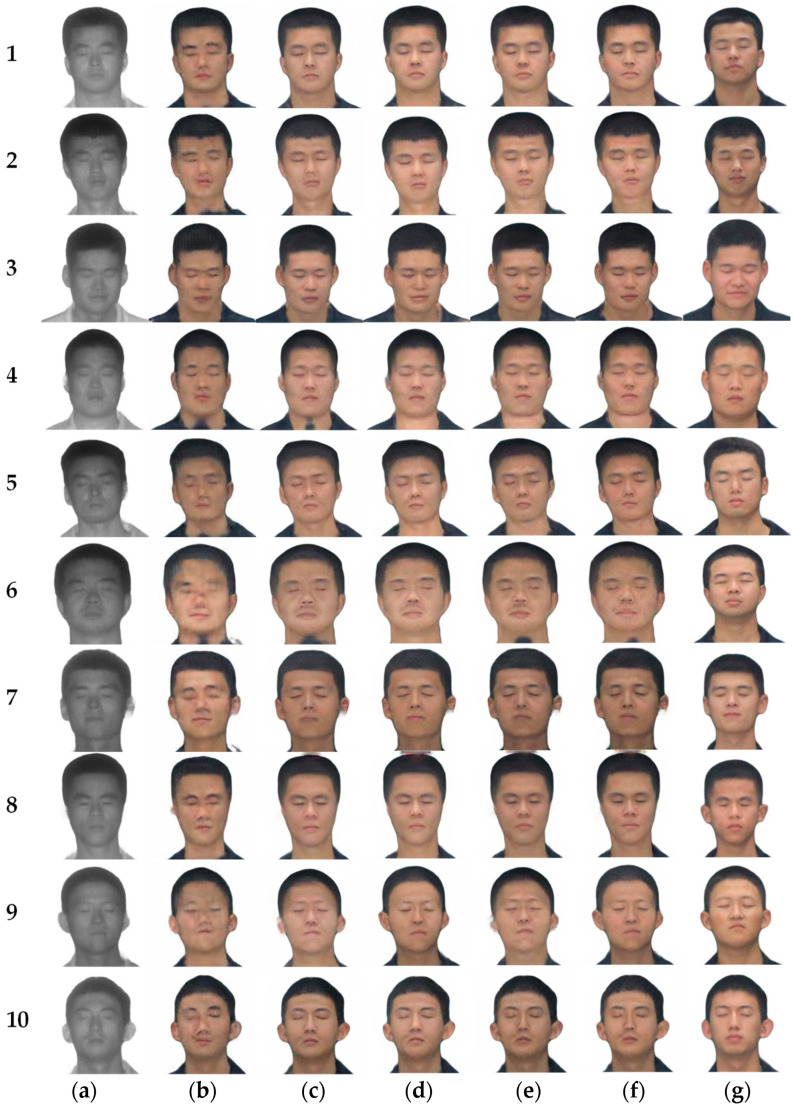
Ablation experiments. (**a**) Laser images. (**b**) CycleGAN. (**c**) CycleGAN+SND. (**d**) CycleGAN+SND+*L_Y_*. (**e**) CycleGAN+SND+*L_identity_*. (**f**) SN-CycleGAN (Ours). (**g**) Ground truth.

In the paper, we use ablation experiments to verify the influence of different variables on the image translation effect, and the translation results are shown in Figure 12. SND represents the name of the improved discriminator, which combines the words spectral normalization and discriminator. $L_{Y}$ represents the reconstruction loss based on the content of the Y channel, and $L_{identity}$ represents the identity loss.

As it can be seen from the figure, these images in Figure 12b have missing facial features and many facial artifacts. The facial features in Figure 12c–f are not lost, but there is a change in skin color. At the same time, there are subtle differences in some details, such as the translation of the sixth line, and the subject’s neck appearing with different degrees of shadowing.

It can be seen from Table 5 that SND is very effective for laser-visible face translation. When using CycleGAN+SND, the quality of translated images is greatly improved compared to CycleGAN. When CycleGAN+SND+*L_Y_*, the FID value decreases by 12.016 compared to CycleGAN. When CycleGAN+SND+*L_identity_*, the FID of the translated image is comparable to that of CycleGAN+SND. When SN-CycleGAN, the FID value is lowest and the quality of the translated images is best in quantification results.

## 4. Laser-Visible Face Recognition 

### 4.1. Improve the Model

The images generated by SN-CycleGAN are good in subjective and FID scores, but the accuracy is lower when face recognition. We improve the SN-CycleGAN network to improve face recognition accuracy.

#### 4.1.1. Generator Structure Based on Feature Retention

**Figure 13:**
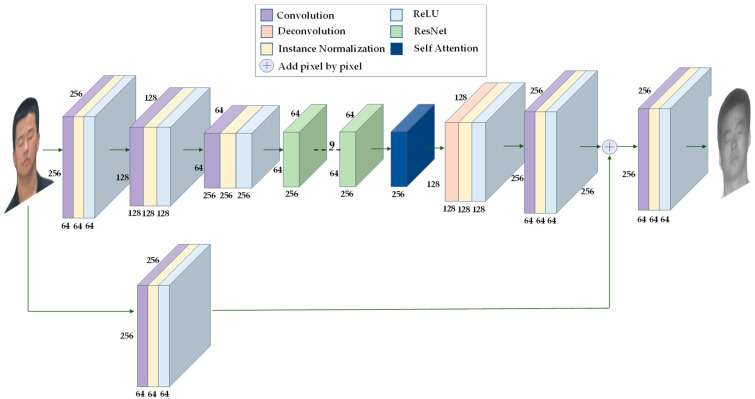
Improved generator structure.

The improvement generator, unlike the original generator, introduces a direct connection between the encoder and decoder. During encoding, the image is compressed after convolution, and this process loses some feature information, which cannot be fully recovered when decoded [29]. We connect the shallow feature map with more detailed information directly to the decoder, which can effectively reduce the loss of detailed information during the generation process. At the same time, we add a self-attention module [30] after ResNet to make the image extraction module pay more attention to important areas. The improved generator structure is shown in Figure 13. The input image generates 64 feature maps, and these feature maps are added to the feature maps after the second convolution in the decoder pixel by pixel. Through this direct connection, shallow feature information is passed directly to the decoder.

#### 4.1.2. Domain Loss Function Based on Triplet Loss Function

**Figure 14:**
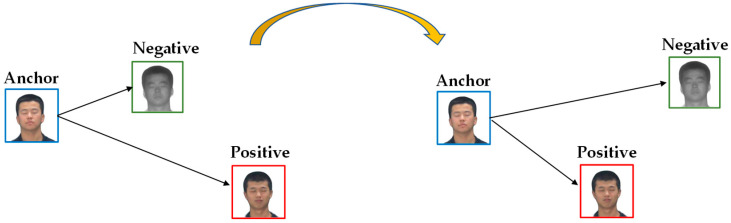
Schematic of a domain loss function based on a triplet loss function.

CycleGAN is prone to the fact that the previous generator learns the error message during training, and the next generator also learns the error message, but the cycle loss function is small. Domain loss functions are introduced for constraint to reduce this problem. A domain loss function based on a triplet loss function [31] is introduced to constrain the style of the image domain. In the early stages of training, the generator does not learn the correct mapping well. The feature distance between same-domain images is smaller than that of cross-domain images. The domain loss function takes advantage of this characteristic to shorten the distance between the same-domain images and increase the distance between the cross-domain images when the feature distance between the same-domain images is greater than that of the cross-domain. The triplet loss function inputs three parameters: Anchor image, Positive image and Negative image. The Anchor image is the base image, the Positive image and the Anchor image are same-domain images, and the Negative image and the Anchor image are cross-domain images. The loss function uses ResNet18 to extract 512-dimensional feature information from three images, and calculates the Euclidean distance between Anchor-Positive and Anchor-Negative images. When the distance of the former is greater than the distance of the latter, the loss function reduces the distance of the former, as shown in Figure 14.

The formula is

(8) $$L_{triplet}(A,P,N)=\max({\left\|D(A)-D(P)\right\|}^{2}-{\left\|D(A)-D(N)\right\|}^{2}+a,0),$$

where *A*, *P*, and *N* represent the Anchor image, the Positive image, and the Negative image, respectively, *a* is constant parameter, and D(·) are feature extraction networks.

Total loss function:

(9) $$\begin{array}{cc} L(G_{LV},G_{VL},D_{L},D_{V}) & =L_{GAN}(G_{LV},D_{V},L,V)+L_{GAN}(G_{VL},D_{L},L,V)\\ & \;+\lambda L_{cyc}(G_{LV},G_{VL})+\alpha L_{identity}(G_{LV},G_{VL})\\ & \;+\beta L_{Y}(G_{LV},G_{VL})+\varphi L_{triplet}(A,P,N) \end{array},$$

where φ is the weight of the domain loss function, set to 2.

### 4.2. Laser-Visible Face Recognition Experiment 

#### 4.2.1. Methods for Evaluating Experimental Results

The translation results of the improved model are evaluated using two methods: subjective observation and objective quantification. Subjective observation relies mainly on the human eyes. Objective quantification mainly uses FID and face recognition accuracy.

Face recognition methods include face verification and face identification. Face verification is a 1:1 process that verifies whether two faces belong to the same identity. Face identification is a 1:N process, which compares the face with the face database to obtain the face with the highest match. For the research of laser-visible face recognition, it is more suitable for face identification methods. With the development of deep learning, visible face recognition algorithms have become very mature. In this paper, FaceNet [31] is used to recognize the face, and Rank-1 is used as the accuracy evaluation index. As an open-source visible face recognition algorithm, FaceNet uses VGGFace2 as the training database and ResNet as the face feature extractor, and finally outputs a 512-dimensional feature vector.

In the test algorithm, the first step is to establish face feature databases. Face data are visible images of 100 collected targets. We use pre-trained MTCNN to detect and capture faces, and use FaceNet to extract the feature information of these faces. We use this facial feature information to build face feature databases. The second step is face recognition. We extract the facial features of the generated image and compare them with the database, and calculate the Euclidean distance between the two in turn. The smallest distance indicates the most likely person. When experimenting with the direct recognition effect of laser faces, MTCNN cannot detect laser faces, and all the laser faces in the test set are manually intercepted and normalized to 160 × 160.

#### 4.2.2. Comparison with Other Models

**Figure 15:**
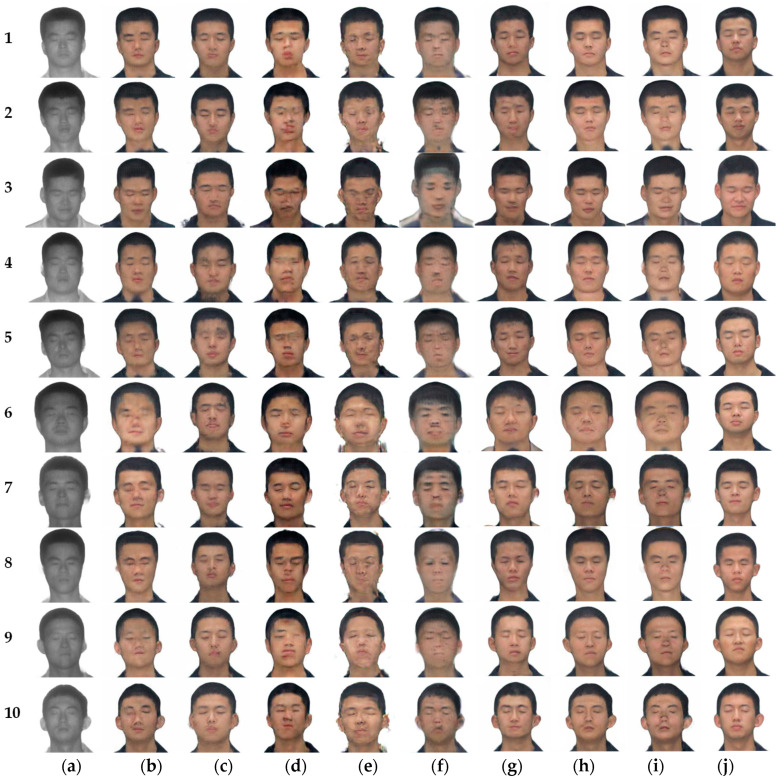
Different methods for Laser-visible facial image translation. (**a**) Laser image. (**b**) CycleGAN. (**c**) Pix2Pix. (**d**) UNIT. (**e**) UGATIT. (**f**) StarGAN. (**g**) DCLGAN. (**h**) SN-CycleGAN. (**i**) Ours. (**j**) Ground truth.

The experimental environment, datasets, and parameters are the same as in Chapter 3. The image translation results under different frameworks are shown in Figure 15, and the FID values are shown in Table 6.

Figure 15b–g have been analyzed in the previous section and will not be repeated here. Subjectively, there is almost no difference in the translation results of Figure 15h,i, but in skin color and some details, Figure 15i does not translate as well as Figure 15h. For example, the shadow on the neck in Figure 15(i-2,i-4), and the collar and skin junction in Figure 15(i-3) are not well treated. Although Figure 15h,i are visually similar, Figure 15i has an FID value of 10.463 higher than Figure 15h does.

When the translated images of CycleGAN, Pix2Pix, UGATIT, StarGAN and DCLGAN are used for face recognition, their Rank-1 face recognition accuracy is almost always 0. UNIT has a face recognition accuracy of 11.5% in Rank-1. When using laser images for face recognition, the face recognition accuracy reaches 57.7%. The face recognition accuracy reaches 53.8% when using translated images of SN-CycleGAN for face recognition. The improved method in this chapter reaches 76.9%, which is 23.1% higher than that of SN-CycleGAN, and 19.2% higher than that of laser face direct recognition. Compared with SN-CycleGAN, the model proposed in this chapter increased the face recognition accuracy by 23.1%, but the FID increased by 10.463. This shows that the model in this chapter is effective for face recognition, but it reduces the image quality.

The laser-visible face recognition based on feature retention proposed in this chapter effectively eliminates the interference of modal differences on face recognition, and the generated image basically conforms to the corresponding real image subjectively and improves the face recognition accuracy objectively.

## 5. Conclusions

We propose a SN-CycleGAN model for laser-visible face image translation, which combines the strengths of spectral normalization and Y channels, and it ensures the network can learn the mapping relationship of laser-visible faces. We use the discriminator composed of spectral normalization, which enhances the stability of the network, improves the convergence speed of the network and reduces face distortion and missing facial features in image translation. The content reconstruction loss function of the Y channel reduces the case of image mismapping. The improved network is compared with the five classic networks subjectively, the image translation results are closer to the visual perception of people, and objectively, the FID score of the improved network is lower. Based on the SN-CycleGAN framework, a laser-visible face recognition scheme based on feature retention is proposed. We use a directly connected structure on the generator to preserve face information, and add a domain loss function to constrain the style between the same domain. Compared with other models, these images generated by the improved model are more in line with human vision, and the face recognition accuracy is improved objectively.

In practical applications, the police obtain the laser face of the criminal in the dark and distant situation, and they convert the laser image into a visible image through the image translation algorithm, and then use the face recognition algorithm to identify the criminal. The experiment employs the method of translation first and then that of recognition, and we can obtain the visible face image of the suspect. At the same time, the translated image can serve as an important clue when a misidentified identity is determined. We propose an algorithm that provides conditions for the application of range-gated laser imaging instrument to security monitoring. Combining the advantages of the visible imaging system during the day ensures that the surveillance system can run all day and improve the work efficiency of the police.

In the actual scenario, we still have problems to further optimize and solve.

1. In practical applications, sometimes, image acquisition equipment cannot collect positive faces, and a large part of images are side faces or obstructed faces. For the face image translation that loses part of the face information, a way to improve the image translation quality is the next research direction.
2. At present, the number of laser datasets is small, and mainly comprises Asian youth. It is necessary to supplement laser data for other races, all ages, and different genders.
3. At present, there are still great difficulties in directly identifying laser face images. Our next step will be on improving the direct recognition accuracy of laser-visible face images.
4. At present, laser face image translation and laser face recognition are only implemented at the algorithm level, and there are still many problems for practical applications. For example, in the image translation task, we ignore the latency of the model while pursuing image quality, which brings new challenges to the real-time translation of the model. In the next step, we will solve the real-time translation problem of the model and the model deployment problem.

## Figures and Tables

**Table 1:** Parameters of visible and laser images acquired.

Parameter	Visible Image	Laser Image
Resolution	5184 × 3456	1024 × 768
Bit depth	24 bit	8 bit

**Table 2:** Hardware or software platform for experimentation.

Hardware or Software Platforms	Parameter
Operating System	Windows 10 Education
GPU	NVIDIAGTX-3090
Memory	24 GB
CPU	Intel(R) Xeon(R) Silver 4116 CPU
CUDA	CUDA11.7
Deep Learning Framework	Pytorch

**Table 3:** FID value of content reconstruction loss under different weights.

Weight	FID
0	37.377
5	39.920
9	42.305
10	36.845
11	42.075
15	39.169
20	37.799
25	38.161

**Table 4:** Image translation quantification results for different frameworks.

Model	FID
CycleGAN	53.747
Pix2Pix50.626UNIT	46.901
UGATIT	94.567
StarGAN	99.443
DCLGAN	37.606
Ours (SN-CycleGAN)	36.845

**Table 5:** Quantitative analysis of ablation experimental results.

Model	FID
CycleGAN	53.747
CycleGAN+SND	37.377
CycleGAN+SND+*L_Y_*	41.731
CycleGAN+SND+*L_identity_*	38.598
SN-CycleGAN (Ours)	36.845

**Table 6:** Laser-visible face image recognition accuracy in Rank-1.

Model	Rank-1/%	FID
Laser image	57.7%	/
CycleGAN	0	53.747
Pix2Pix	0	50.626
UNIT	11.5%	46.901
UGATIT	0	94.567
StarGAN	0	99.443
DCLGAN	0.077%	37.606
SN-CycleGAN	53.8%	36.845
Ours	76.9%	47.308

## Data Availability

Not applicable.

## References

[1] Deng J., Guo J., Yang J., Xue N., Kotsia I., Zafeiriou S. (2022). ArcFace: Additive Angular Margin Loss for Deep Face Recognition. arXiv.

[2] Youchen F., Baolin L., Mingyu Q., Huichao G., Mingqian W. (2022). Application of range-gated imageing in UAV recognition and classification. Laser Infrared.

[3] Youchen F., Hongli Z., Huayan S., Huichao G., Yanzhong Z. (2015). Study on simulation of laser active imagimg under atmospheric conditions. Laser Infrared.

[4] Tian S., Cheolkon J., Qingtao F., Qihui H. (2019). NIR to RGB Domain Translation Using Asymmetric Cycle Generative Adversarial Networks. IEEE Access.

[5] Xu J. (2022). Research on polarimetric facial thermal-visible image translation. Master’s Thesis.

[6] Mei K., Mei Y., Patel V.M. (2022). Thermal to Visible Image Synthesis under Atmospheric Turbulence. arXiv.

[7] Shengchong Z., Shuwei T., Haibo Z. (2009). Laser Active Imaging Tecchnology. Electro. Opt. Technol. Appl..

[8] Shouzeng W., Feng S., Xin Z. (2008). Development of laser illuminating range-gated imaging technique. Infrared Laser Eng..

[9] Mingyu Q., Youchen F., Huichao G., Mingqian W. (2022). Application of Improved CycleGAN in Laser-Visible Face Image Translation. Sensors.

[10] He Z., Patel V.M., Riggan B.S., Hu S. Generative Adversarial Network-based Synthesis of Visible Faces from Polarimetric Thermal Faces. Proceedings of the 2017 IEEE International Joint Conference on Biometrics (IJCB).

[11] Zhongling W., Zhenzhong C., Feng W. (2018). Thermal to Visible Facial Image Translation Using Generative Adversarial Networks. IEEE Signal Process. Lett..

[12] Cunjian C., Arun R. Matching Thermal to Visible Face Images Using a Semantic-Guided Generative Adversarial Network. Proceedings of the 2019 14th IEEE International Conference on Automatic Face & Gesture Recognition (FG 2019).

[13] Babu K.K., Dubey S.R. (2020). PCSGAN: Perceptual Cyclic-Synthesized Generative Adversarial Networks for Thermal and NIR to Visible Image Transformation. Neurocomputing.

[14] Karras T., Laine S., Aittala M., Hellsten J. (2020). Analyzing and Improving the Image Quality of StyleGAN. arXiv.

[15] Linmiao H. (2020). Research on Key Technologies of Face Image Enhancement and Recognition Based on Shortwave-Infrared Imaging System. Ph.D. Thesis.

[16] Qiujuan P., Yan Y., Liang C., Yi W. (2010). Eye safety analysis for 400–1400 nm pulsed lasers systems. Laser Infrared.

[17] Zhu J.-Y., Park T., Isola P., Efros A.A. Unpaired Image-to-Image Translation using Cycle-Consistent Adversarial Networks. Proceedings of the 2017 IEEE International Conference on Computer Vision (ICCV).

[18] Isola P., Zhu J., Zhou T., Efros A.A. Image-to-Image Translation with Conditional Adversarial Networks. Proceedings of the 2017 IEEE Conference on Computer Vision and Pattern Recognition (CVPR).

[19] Goodfellow I.J., Pouget-Abadie J., Mirza M., Xu B., Warde-Farley D., Ozair S., Courville A., Bengio Y. Generative Adversarial Nets. Proceedings of the Advances in Neural Information Processing Systems 27 (NIPS 2014).

[20] Arjovsky M., Chintala S., Bottou L.E. (2017). Wasserstein GAN. arXiv.

[21] Takeru M., Toshiki K., Masanori K., Yuichi Y. (2018). Spectral Normalization for Generative Adversarial Networks. arXiv.

[22] Xia W., Huiying X., Xinzhong Z. (2022). A text-to-image model based on the two-phase stacked generative confrontation networks with spectral normalization. Comput. Eng. Sci..

[23] Linmiao H., Yong Z. (2020). Facial Image Translation in Short-Wavelength Infrared and Visible Light Based on Generative Adversarial Network. Acta Opt. Sin..

[24] Lezama J., Qiu Q., Sapiro G. (2016). Not Afraid of the Dark: NIR-VIS Face Recognition via Cross-spectral Hallucination and Low-rank Embedding. arXiv.

[25] Kim J., Kim M., Kang H., Lee K. (2019). U-GAT-IT: Unsupervised Generative Attentional Networks with Adaptive Layer-Instance Normalization for Image-to-Image Translation. arXiv.

[26] Choi Y., Choi M., Kim M., Ha J.-W., Kim S., Choo J. StarGAN: Unified Generative Adversarial Networks for Multi-Domain Image-to-Image Translation. Proceedings of the IEEE Conference on Computer Vision and Pattern Recognition (CVPR), Salt Lake City.

[27] Liu M.-Y., Breuel T., Kautz J. (2018). Unsupervised Image-to-Image Translation Networks. arXiv.

[28] Han J., Shoeiby M., Petersson L., Armin M.A. (2021). Dual Contrastive Learning for Unsupervised Image-to-Image Translation. arXiv.

[29] Xu J., Lu K., Shi X., Qin S., Wang H., Ma J. (2021). A DenseUnet generative adversarial network for near-infrared face image colorization. Signal Process..

[30] Zhang H., Goodfellow I., Metaxas D., Odena A. (2019). Self-Attention Generative Adversarial Networks. arXiv.

[31] Schroff F., Kalenichenko D., Philbin J. (2015). FaceNet: A Unified Embedding for Face Recognition and Clustering. arXiv.

